# Conservative Gynecology

**Published:** 1894-02

**Authors:** 


### EDITORIAL DEPARTMENT.

F. E. DANIEL, M. D., Editor.
S. E. HUDSON, M. D., Managing Editor.
A. J. SMITH. M. D., Galveston, Associate Editor.

EDITORIAL STAFF:

PROF. J. E. THOMPSON, M. D., Texas Medical College, Galveston; Surgery.
PROF. WM. KEILLER, M. D., Texas Medical College, Galveston; Obstetrics and Gynecology.
PROF. DAVID CERNA, M. D., Texas Medical College, Galveston; Therapeutics.
PROF. A. J. SMITH, M. D., Texas Medical College, Galveston; Medicine
DR. ISADORE DYER, Tulane University; Dermatology.
DR. R. H. L. BIBB, Saltillo, Mexico; Foreign Correspondent.
DR. ROBT. MORRIS, Charity Hospital, N. O.; Clinical Reports

### CONSERVATIVE GYNECOLOGY.

That which is not true is false, and half truth is falsehood's most dangerous shape. The common knowledge of the profession should be the court of final appeal in questions of medical truth.—*L. F. Bishop, in Medical Record, Jan. 13, 1894.*

Prof. William Goodell, of Philadelphia, than whom no living authority on gynecology stands higher or is better known in America as well as in Europe, has recently published two articles entitled: "The Effect of Castration on Women, and other Problems in Gynecology," and "The Great Medical Error of the Day," which have appeared in the *Medical News* and the *University Medical Magazine*, respectively. Both articles run in the same channel of thought, and a careful perusal of them reveals the sad fact that the profession is on the verge almost of criminal abuse regarding the surgical treatment of female diseases.

It must not be forgotten that in medicine, as in almost everything else, fads are apt to creep in, to maintain a firm hold (for a time at least) on the profession, even at the sacrifice of scientific truth or common sense, until new ones come to break the magic spell of the old ones by virtue of the falsity of these. And it is among the younger members of the profession, of course, that such fads most flourish. In the meantime, suffering humanity is the victim; and it seems as if experience were unable to cope successfully with the danger of the dogmatic principle, "*magister dixit.*" The disastrous failure of the tuberculin craze, for instance, should be a lasting lesson to the advocates of the *new* in the healing art, and especially to those who, at one single flight would wish to reach the pinnacle of fame and glory!

In support of the fact that there is a tendency on the part of the profession to follow a fad, (and with reference especially to gynecological practice), Prof. Goodell most pertinently remarks: "In the treatment of the diseases of women at the present time, there seems to me to be a tendency to lay too much stress upon lesions of the reproductive organs. Too little heed is, therefore, given to the nerve-element of woman's diseases, and as a consequence, the surgical feelers and antennæ of the medical profession, always too keenly sensitive, vibrate vehemently at the approach of an ailing woman. This trend of the profession, to appeal to the knife as the great panacea for woman's diseases, is seen everywhere. It prevails alike in city, town, village and hamlet. It asserts itself in every medical discussion, and stands out in bold relief upon the pages of every medical journal. It has caused many needless sexual mutilations and unnecessary operations, and it is, in my opinion, the great medical error of the day."

We are convinced that gynecological surgery, all in all, has done, and continues to do more harm than good. Fashion, mingled with ignorance on the one hand, and with *too much* knowledge perhaps upon the other hand, has brought about a most unfortunate state of affairs, and wrought much evil. There is, we firmly believe, entirely too much mutilation being done upon women, unnecessarily, and for ills many of them imaginary or attributable to some derangement of that complex machinery, *the nervous system.* To use a homely simile, it may be said that the nervous system constitutes a safe that up to the present time even the most expert scientific burglars have been unable to unlock completely. How ignorant we are of the physiology of the nervous system !

To reiterate, it seems to us that more misery than good has been accomplished by reckless, often unwarrantable abdominal and pelvic surgery, especially at the hands of ambitious, hasty and injudicious young practitioners, not to say at the hands also of the fashionable gynecologist or more experienced surgeon. First, Cæsarian section, or laparotomy, or *cœliotomy* more modernized, then, ovariotomy, and *now* symphisiotomy (*revived!*) What next?

Fortunately, our individual conscience is not haunted by the unwelcome remembrance of having given pain, instead of relief, by surgical intervention in the female disorders that have come under our limited observation. We do not regret not to have used the knife in the treatment of diseases which we then suspected, and have reason to believe now, were not due mainly to womb or ovarian trouble. We remember distinctly three cases of female disease, apparently hopeless, occurring in our practice (in each one of which operation was advised by eminent gynecologists, but fortunately flatly refused by the patients themselves or some member of the family), in which recovery ultimately ensued after these cases had been left severely alone.

Of course, we are also satisfied that there are extraordinary cases in which an operation is absolutely necessary, the means of saving life, perhaps, but we likewise believe that these cases are few and far between. Thus Prof. Goodell says: "I must confess to becoming far less inclined than formerly to operate on trifling lesions of the reproductive organs, and especially on small tears of the perineum, the repair of which is painful, unnerving, and generally of doubtful expediency. I have become very skeptical of the influence of such lesions upon the general health, and have come to the belief that, even in bad cases, it is greatly overrated. In my experience the mistake usually made in these cases is that of attributing to the lacerations the mock uterine symptoms of nerve prostration. About this there can be no error, for I have over and over again, without any surgical treatment whatever, cured of all their ailments patients who had been sent to me for the very purpose of undergoing some operation on the womb, on the perineum, or even on the ovaries themselves."

There can be scarcely any doubt but that the nervous system, more so than in the case of man, plays a most important part in female diseases. And it is precisely our ignorance of the physiology, and hence of the pathology of the nervous system, that often leads us astray. As our eminent author wisely puts it: "Again, what is very perplexing, uterine symptoms are by no means always present in cases of utetine disease; and, what is still more bewildering, when so-called uterine symptoms are then present, they need not necessarily come from uterine disease. The nerves are mighty mimes, the greatest mimics, and they cheat us by their realistic personations of the organic disease, and especially by their life-like imitations of uterine disease. Hence it is that even seemingly urgent uterine symptoms may be merely nerve-counterfeits of uterine disease. In fact, the time has come when we must give up the belief, which with many amounts to a creed, that woman is woman because she has a womb, and that the womb is at the bottom of nearly every female ailment.''

Thoroughly in accord with the sound views of Prof. Goodell, we wish to be placed on record as protesting most earnestly against the indiscriminate use of the knife in the treatment of diseases of women. We plead for conservative gynecology. But we cheerfully cede the floor to the learned teacher and writer: ''Just as headache does not necessarily mean brain disease, so ovary-ache, groin-ache and backache do not necessarily mean ovarian disease. Nerve-strain and these aches are, it is true, correlative, but the middle term which connects them is merely a disturbance in the circulation. Yet, time and again—and I say this deliberately—have ladies been sent to me to have their ovaries taken out, when the whole mischief had started from some mental worry. Their ovaries were sound, but their nerves were not, and no operation was needed for their recovery. The physician of the present day is too apt to jump from any distinctly female ache to an ovarian conclusion without the delay of any misgivings. The ache is in the back, then, he argues, it probably is ovarian; it is in the groin, then, of course, it is ovarian; it is in the head, but extremes meet, and surely it comes from the ovaries. I, indeed, have seen a painful nose, and also a red one, attributed to the ovaries, and treated canonically by the hot vaginal douche and uterine applications. From this widespread bias and pernicious haste, the removal of the ovaries has degenerated into a busy industry by which, in city and in country, many women have been and are being mutilated, both needlessly and on the slightest provocation.'' * * * ''The riper my experience, the more am I convinced that, in the treatment of woman's diseases, the possibility of a nerve-origin, or of a nerve-complication should be the *fore*-thought and not the *hind*-thought of the physician.''

Were it in our power, we should have *every book* on gynecology extant and to be published hereafter, prefaced with the first six and last paragraphs of the excellent article ''The Great Medical Error of the Day.'' Certainly those paragraphs are pregnant with sound thought and most useful counsel; they, in fact, contain in a few words what most of the so-called scientific treatises on gynecology *do not*. With his authoritative statements, wielding a facile and trenchant pen, and based on a long and useful experience, an experience unsullied by dogmatism, wedded alone to common sense, to sound and unbiased judgment, we sincerely trust that Prof. Goodell will continue to wage war mercilessly on reckless gynecological practice as detrimental to the welfare of the gentler sex.

Many, many cases of female complaint, we are confident, not only do never reach the eager publishing gynecologist, but do not even come under the observation of the operating surgeon. This being the case, are we justified in judging of the necessity or the expediency of mutilating alone from the comparatively small number of successful (?) cases found recorded in current medical literature? The silent majority in the medical profession (properly conservative) is and shall continue to be, even though the language be paradoxical, a disturbing element to the otherwise clear and peaceful conscience of the gynecological surgeon.

It is high time that the fascinating temple of excessive specialism in medical practice, should close its doors, and that the over-enthusiastic advocates of the knife, especially, should remove their mask and listen to the voice of reason and of nature. After all, nature is the best of physicians. Let nature, then (nature to which personal experience and science itself are subservient), open the pathway, and truth will appear, like the radiant Phœbus before the dying dawn, gorgeously arrayed in all its majesty and spendor.

Some one has said, wisely, that "truth travels slowly, and error is hard to rout," yet, this fact should discourage no one in the pursuit of a lofty cause, in the noble search for truth and right. We beseech Prof. Goodell, therefore, to continue letting us have the benefit of his wise counsel in matters gynecological. It will be, we are sure, of incalculable service to suffering humanity, particularly to helpless woman, that female Lear of gynecological tragedy, so to speak, a creature, indeed, "more sinned against than sinning!" Let his honored and respected name, as it is already deservedly through his excellent work so far accomplished, continue to be a household word wherever female disease is considered, that, more strengthened still by virtue of his riper and riper experience, it may be handed down to posterity as belonging by rights to one of the benefactors of the race. The structure he will have erected in the end, will constitute a monument more durable, indeed, than the pyramids of Egypt; and his glorious name, standing out in bold relief upon such a monument, and surrounded by the halo of immortality, shall be gratefully sounded by the tongue of man even long after those of many now famous mutilators of our species shall have been forever erased from the tablets of human thought!

